# Recurrent Optic Neuritis With MRI-Negative Myelopathy in Myelin Oligodendrocyte Glycoprotein Antibody-Associated Disease (MOGAD)

**DOI:** 10.7759/cureus.100005

**Published:** 2025-12-24

**Authors:** Karishma Harrilal-Maharaj, Branimir Nevajda

**Affiliations:** 1 Internal Medicine, Basildon University Hospital, Basildon, GBR; 2 Stroke, Mid and South Essex NHS Foundation Trust, Basildon, GBR

**Keywords:** mog antibody, mog antibody-associated disease, mri-negative myelitis, optic neuritis, plasma exchange, steroid-refractory

## Abstract

Myelin oligodendrocyte glycoprotein antibody-associated disease (MOGAD) is an inflammatory demyelinating disorder that frequently presents with optic neuritis or myelitis, although early diagnostic evaluation may be challenging when neuroimaging is normal despite significant neurological deficits. We describe a 29-year-old woman who initially developed unilateral optic neuritis, followed one month later by sequential contralateral involvement and new sensory disturbances. She subsequently presented with acute bilateral lower-limb weakness, brisk reflexes, and lumbosacral sensory disturbance, although spinal MRI remained normal. Progressive neurological decline prompted treatment with high-dose IV corticosteroids and plasma exchange. MOG-IgG later returned positive, confirming MOGAD. The patient experienced gradual recovery of left-eye color perception and lower-limb strength, whereas right-eye visual acuity remained profoundly reduced. This case underscores the importance of recognizing recurrent or bilateral optic neuritis as a potential harbinger of MOGAD, appreciating that clinically evident myelopathy may occur with normal MRI, and initiating early neurology involvement and treatment escalation to prevent irreversible disability.

## Introduction

Myelin oligodendrocyte glycoprotein antibody-associated disease (MOGAD) is an immune-mediated demyelinating disorder of the CNS characterized by optic neuritis, myelitis, and, less commonly, acute disseminated encephalomyelitis [1-3]. Although it shares overlapping features with multiple sclerosis (MS) and aquaporin-4-positive neuromyelitis optica spectrum disorder (AQP4-NMOSD), MOGAD represents a distinct pathological and clinical entity. Patients typically demonstrate more pronounced optic nerve involvement, a monophasic or relapsing course, and a characteristic response to corticosteroids, while lacking the chronic neurodegenerative features associated with MS [1-3].

Diagnosing MOGAD can be challenging in the acute setting. MRI abnormalities may be subtle or even absent despite clinically significant inflammation, particularly in cases of early myelitis [4,5]. This can lead to diagnostic uncertainty, especially when symptoms evolve rapidly or do not conform to classical MS or NMOSD patterns. Recurrent or sequential optic neuritis, bilateral involvement, steroid responsiveness, and disproportionately severe visual loss should raise suspicion for MOGAD even when neuroimaging is nondiagnostic [4,6].

We describe a young woman who developed sequential bilateral optic neuritis followed by clinically evident myelopathy in the context of a normal spinal MRI. This case highlights the diagnostic pitfalls associated with MRI-negative MOGAD and underscores the importance of a clinically driven approach, supported by timely antibody testing and early therapeutic intervention. While isolated reports of MRI-negative myelitis exist, the combination of rapidly sequential bilateral optic neuritis followed shortly thereafter by clinically significant but radiologically silent myelopathy is relatively uncommon in adults. This pattern can create substantial diagnostic uncertainty, particularly early in the disease course.

By presenting this case, we aim to draw attention to this under-recognized phenotype, illustrate the limitations of spinal MRI in early MOGAD, and reinforce the need for clinical suspicion to prompt early antibody testing and treatment escalation. The objective of this report is therefore to support clinicians in identifying similar presentations and preventing delays that may contribute to poorer visual or neurological outcomes.

## Case presentation

A 29-year-old woman with a history of migraines presented to ophthalmology in late August 2025 with blurred vision in her right eye. She also reported a one-year history of intermittent paresthesia in both hands. On initial ophthalmic assessment, the right optic disc appeared normal, with no disc edema (grade 0), consistent with retrobulbar optic neuritis. Color vision testing using Ishihara plates was mildly reduced in the right eye (22/24) and normal in the left (24/24), with no relative afferent pupillary defect at this stage. Extraocular movements were full, with pain reported on eye movement in multiple directions, most prominent on horizontal gaze.

**Figure 1:**
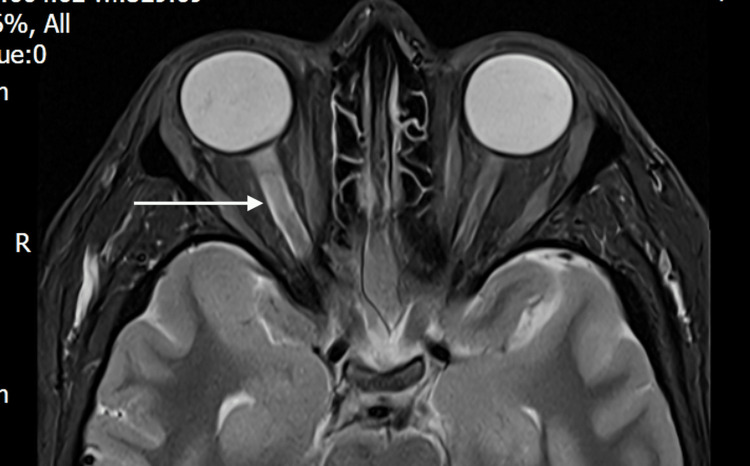
Axial T2-weighted fat-suppressed MRI of the orbits, performed shortly after symptom onset, showing hyperintensity and swelling of the right optic nerve (arrow) consistent with acute optic neuritis, with no additional intracranial abnormalities

MRI of the brain and orbits performed one week later demonstrated high signal within the right optic nerve, consistent with optic neuritis (Figure 1), with no additional abnormalities. There was no evidence of optic nerve sheath or perineural enhancement, no optic chiasmal involvement, and no intracranial parenchymal lesions. She was discharged without corticosteroid therapy.

She re-presented to the ED one month after her initial presentation with sudden visual loss in the left eye, accompanied by painful eye movements and a severe headache resembling her typical migraine. Eye pain preceded the visual loss and was again most noticeable on horizontal gaze. Examination revealed no perception of light in the right eye and a visual acuity of 6/24 in the left eye. A right relative afferent pupillary defect was now present, and color vision was markedly reduced in the right eye. She also described tingling in the right upper limb and intermittent facial paresthesia. She was initially reviewed by the stroke team, with no focal cortical or brainstem signs identified, and was subsequently assessed in the eye casualty, where left-eye visual acuity, color vision, pupillary responses, and fundoscopic findings were unchanged from the earlier assessment. Owing to her progressive symptoms, she was admitted for medical evaluation. A repeat MRI of the brain and orbits demonstrated persistent right optic nerve swelling and a subtle high signal in the posterior segment of the left optic nerve; the brain parenchyma remained normal. No perineural or optic nerve sheath enhancement was identified. She received three days of IV methylprednisolone followed by a five-day oral taper.

In early October, approximately six weeks after her initial presentation, she attended another ED within the same trust, reporting new bilateral lower-limb weakness, intermittent tingling, and “shock-like” sensations in both legs, more prominent in a lumbosacral distribution. She also noted transient numbness of the posterior left thigh. Examination revealed mild bilateral lower-limb weakness and brisk reflexes, with preserved coordination and no sensory level.

An MRI of the brain and entire spine performed during this admission showed interval improvement of the right optic neuritis but no signal abnormality within the spinal cord. Lumbar puncture demonstrated elevated protein (0.77 g/L) with normal cell counts and negative oligoclonal bands; viral and bacterial studies were also negative. This CSF profile, characterized by elevated protein with normal cell counts and negative oligoclonal bands, in the context of sequential optic neuritis and clinically evident but MRI-negative myelopathy, was felt to be more supportive of MOGAD than MS or AQP4-positive NMOSD.

Due to ongoing neurological deterioration, neurology recommended a further course of IV methylprednisolone and arranged a consultation with a tertiary neuroscience center, which accepted her for plasma exchange (PLEX). She underwent five sessions of PLEX between October 13 and 17, 2025, and was discharged on a tapering course of oral prednisolone.

Serum MOG-IgG and AQP4-IgG were sampled during the tertiary admission, after corticosteroid treatment, and prior to initiation of PLEX. AQP4-IgG was negative, while MOG-IgG returned positive at a titer of 1:10 using a cell-based assay, confirming the diagnosis of MOGAD. At follow-up in early November, she reported gradual improvement in left-eye color perception and lower-limb strength, although right-eye visual acuity remained profoundly reduced. She also described a further episode of back pain and transient weakness despite ongoing steroid therapy.

A chronological summary of the patient’s clinical course, investigations, and management is provided in Table 1.

**Table 1:** Chronological summary of the patient’s clinical course, investigations, and management during the evaluation and treatment of MOGAD AQP4, aquaporin-4; MOGAD, myelin oligodendrocyte glycoprotein antibody-associated disease; OCB, oligoclonal bands; PLEX, plasma exchange

Time point	Key clinical features	Investigations	Management
Late August 2025	Right-eye visual blurring; intermittent bilateral hand paresthesia	MRI brain/orbits: right optic neuritis; no intracranial lesions	No corticosteroids
September 2025	Sequential left optic neuritis; painful eye movements (horizontal gaze); severe visual loss	MRI brain/orbits: persistent right optic neuritis; subtle left optic nerve involvement; no perineural enhancement	IV methylprednisolone ×3 days, oral taper
Early October 2025	Bilateral lower-limb weakness; paresthesia; shock-like lumbosacral sensations; hyperreflexia	MRI brain and spine: no spinal cord lesions; CSF: elevated protein, normal cell count, OCB negative	IV methylprednisolone; tertiary referral
Mid-October 2025	Progressive neurological symptoms	Serum MOG-IgG positive (1:10); AQP4-IgG negative	PLEX ×5 sessions
Early November 2025	Improved left-eye color vision and lower-limb strength; persistent right-eye visual loss	-	Ongoing oral steroid taper

## Discussion

MOGAD encompasses a spectrum of inflammatory demyelinating disorders characterized by optic neuritis, myelitis, or acute disseminated encephalomyelitis [1-3]. Unlike MS or AQP4-NMOSD, MOGAD often demonstrates steroid responsiveness, less chronic neurodegeneration, and distinct radiological patterns [1-3]. Optic neuritis represents the most common presentation, occurring in up to 60-80% of cases, frequently with bilateral or sequential involvement and often associated with substantial optic disc edema [4-6]. Myelitis is similarly well recognized, but MRI-negative myelopathy remains an underappreciated diagnostic pitfall [4,5].

MRI-negative myelopathy in MOGAD

Although spinal cord involvement typically produces T2-hyperintense lesions on MRI, up to 10-15% of patients with MOGAD myelitis may demonstrate normal early spinal imaging despite clear clinical signs of cord dysfunction [4,5]. Proposed explanations include transient inflammatory changes below the threshold of radiological detection, reversible conduction block, or very early disease presentation before radiological evolution. These cases highlight the limitations of relying solely on MRI for diagnosis in demyelinating conditions.

Our patient exhibited bilateral lower-limb weakness, hyperreflexia, and lumbosacral sensory disturbance, findings strongly suggestive of myelopathy, yet her spinal MRI remained normal. The coexistence of recent optic neuritis further strengthened the suspicion of an inflammatory demyelinating process. This case reinforces that a normal spinal MRI does not exclude clinically significant myelopathy in MOGAD, and clinicians should maintain a high index of suspicion when neurological findings and recent optic neuritis coexist.

Diagnostic complexity and evolving clinical course

MOGAD frequently evolves in a stepwise pattern, with individual relapses affecting different regions of the CNS [1,3,6]. In this case, the patient’s clinical course progressed from unilateral optic neuritis to sequential bilateral involvement, then to acute myelopathic symptoms within a six-week period. Early symptoms lacked widespread neurological involvement, and brain MRI remained normal, which can obscure the broader diagnosis [4,5]. Indeed, early presentations may mimic MS, idiopathic optic neuritis, or even migraine-associated visual disturbance, particularly in young adults [4-6]. CSF findings may assist in differentiation, as MOGAD often shows elevated protein with negative oligoclonal bands, unlike MS [7,8]. In this case, MOG-IgG was detected using a cell-based assay at a low positive titer (1:10), which was interpreted as clinically meaningful in the context of recurrent optic neuritis, MRI-negative myelopathy, and recent corticosteroid exposure, which is recognized to reduce circulating antibody titers.

Studies have shown that sequential or bilateral optic neuritis, severe visual loss, and pain with eye movement should prompt early consideration of MOGAD, even in the absence of definitive MRI findings [6]. Early neurology involvement is crucial, particularly when symptoms evolve rapidly or when atypical features such as bilateral optic neuritis or steroid-responsive relapses are present. This case demonstrates precisely how the clinical picture, rather than imaging alone, should guide investigation and acute management.

Treatment implications

High-dose corticosteroids remain the first-line treatment for acute MOGAD attacks, with many patients showing rapid improvement [9,10]. However, up to one-third of patients may experience steroid-refractory disease requiring escalation to PLEX or IV immunoglobulin [11]. The decision by the tertiary center to initiate PLEX before the MOG-IgG result returned aligns with recommendations for severe or progressive optic neuritis and myelitis, particularly when visual acuity is significantly impaired [11,12].

Our patient experienced partial neurological recovery following PLEX, consistent with reported outcomes in the literature [12], but persistent right-eye visual loss underscores that delayed recognition or incomplete early treatment may be associated with poorer visual prognosis [9,10]. Long-term relapse rates in MOGAD may reach 30-50%, and recurrent attacks can lead to cumulative disability, highlighting the need for ongoing specialist follow-up and consideration of maintenance immunotherapy in selected patients [13].

Comparison of MOGAD and AQP4-positive NMOSD

Although MOGAD and AQP4-positive NMOSD can present with overlapping features such as optic neuritis and longitudinally extensive myelitis, they represent distinct autoimmune disorders with important diagnostic and therapeutic implications [2,3]. NMOSD is driven by AQP4-IgG antibodies targeting astrocytes, whereas MOGAD reflects an oligodendrocytopathy with immune targeting of myelin oligodendrocyte glycoprotein [2,3]. Optic neuritis in NMOSD often causes profound and irreversible visual impairment with frequent chiasmal or optic tract involvement, while MOGAD more commonly presents with bilateral or sequential optic neuritis, marked optic disc edema, and generally better visual recovery following corticosteroid treatment [4-6,9,10]. Myelitis in NMOSD is typically severe, longitudinally extensive, and associated with early sphincter dysfunction and high relapse risk, whereas MOGAD myelitis may demonstrate better functional outcomes and can be MRI-negative in the early disease course [4,5]. Treatment responses also differ significantly: MOGAD generally shows a strong response to corticosteroids, and PLEX often yields substantial recovery in severe attacks, while NMOSD relapses frequently leave residual deficits despite aggressive therapy [9-12]. Long-term immunosuppression is essential for all AQP4-positive NMOSD patients due to its highly relapsing nature, whereas maintenance therapy in MOGAD is individualized based on relapse frequency, attack severity, and functional outcomes [11-13]. A comparison of the key clinical and treatment differences between MOGAD and AQP4-positive NMOSD is summarized in Table 2.

**Table 2:** Key clinical and treatment differences between MOGAD and AQP4-NMOSD, including typical patterns of optic neuritis, myelitis, treatment response, and long-term management AQP4, aquaporin-4; AQP4-NMOSD, aquaporin-4-positive neuromyelitis optica spectrum disorder; MOGAD, myelin oligodendrocyte glycoprotein antibody-associated disease; PLEX, plasma exchange

Feature	MOGAD	AQP4-NMOSD
Primary target	Myelin oligodendrocyte glycoprotein (oligodendrocytes)	AQP4 (astrocytes)
Optic neuritis pattern	Often bilateral/sequential; disc edema; better recovery	Often severe, profound, sometimes chiasmal; poorer recovery
Myelitis	May be MRI-negative early; good recovery in many cases	Typically longitudinally extensive; severe, with early bladder involvement
Steroid responsiveness	Typically strong, rapid improvement [9,10]	Often incomplete; PLEX frequently required [11,12]
Effect of PLEX	Substantial visual/neurological recovery [11,12]	Helpful but often with residual disability [11,12]
Long-term immunotherapy	Individualized (monophasic vs. relapsing) [11-13]	Essential in all AQP4-positive patients [11-13]
Relapse pattern	Monophasic or relapsing; relapse risk variable [13]	Highly relapsing, often severe [11-13]

Broader clinical and system-level considerations

This case also illustrates the importance of robust clinical pathways for inflammatory optic neuropathies. Clear guidance on when to involve neurology, when to initiate extended antibody testing, and when to escalate treatment may reduce delays in diagnosis. Given that MRI-negative myelopathy and evolving optic neuritis can lead to diagnostic uncertainty, reliance on multidisciplinary decision-making rather than radiological findings alone is essential.

Patient perspective

The patient expressed significant frustration at the continued, low-grade progression of her symptoms, particularly the persistent visual disturbance in her right eye. She described how this impairment limits her ability to carry out day-to-day activities, especially tasks requiring prolonged computer use, resulting in frequent breaks and reduced confidence at work. She also reported ongoing anxiety about the risk of relapse and the possibility of losing vision in her left eye, which has been a major source of psychological distress.

She felt that certain aspects of her early care could have been improved. In her view, a more detailed assessment of her symptoms, particularly differentiating new neurological features from her usual migraine-related complaints, might have prompted earlier treatment. She also felt that clearer explanations regarding the significance of optic neuritis and the potential differential diagnoses, including conditions such as NMOSD or MOGAD, would have enabled her to advocate more strongly for urgent therapy at the time.

Looking back, she recognized that several early symptoms, such as stiffness and weakness in her legs and back, intermittent paresthesia, fatigue, and fluctuating visual blurriness, were more significant than she had initially appreciated, but she had attributed them to migraines or lifestyle factors. She reflected that greater awareness of the potential implications of optic neuritis may have influenced how she interpreted and escalated these symptoms.

Her reflections highlight the emotional, functional, and psychological impact of MOGAD and underscore the importance of early recognition, clear communication, and patient-centered care, not only in inflammatory neuropathies but across all medical conditions.

## Conclusions

This case illustrates the diagnostic challenges posed by MOGAD, particularly when evolving neurological symptoms occur in the context of normal spinal imaging. Recurrent or bilateral optic neuritis should alert clinicians to the possibility of MOGAD, even when MRI findings appear limited or nondiagnostic. The development of clinically evident myelopathy despite a normal spinal MRI underscores the need to prioritize the clinical examination over radiological appearances in early disease. Timely MOG-IgG testing and early escalation to treatments such as high-dose corticosteroids and PLEX are essential to prevent irreversible visual and neurological morbidity. Ultimately, this case reinforces the importance of recognizing atypical inflammatory demyelinating presentations and adopting a clinically driven approach to diagnosis and management.
